# Comparison of pegaspargase with concurrent radiation *vs*. P-GEMOX with sequential radiation in early-stage NK/T-cell lymphoma

**DOI:** 10.32604/or.2024.057065

**Published:** 2025-03-19

**Authors:** DEMEI FENG, SHENRUI BAI, GUANJUN CHEN, BIBO FU, CAILU SONG, HAILIN TANG, LIANG WANG, HUA WANG

**Affiliations:** 1 State Key Laboratory of Oncology in South China, Guangdong Provincial Clinical Research Center for Cancer, Sun Yat-sen University Cancer Center, Guangzhou, 510060, China; 2 Department of Hematology, Beijing Tongren Hospital, Capital Medical University, Beijing, 100730, China

**Keywords:** Natural killer/T-cell lymphoma (NKTCL), Concurrent radiation therapy, Pegaspargase, Propensity score matching

## Abstract

**Objectives:**

The optimal treatment strategy for early-stage natural killer/T-cell lymphoma (NKTCL) remains unclear. This study aimed to evaluate and compare the clinical outcomes and adverse events (AEs) associated with two treatment regimens for early-stage NKTCL: pegaspargase with concurrent radiation therapy (P+CCRT) and pegaspargase, gemcitabine, and oxaliplatin (P-GEMOX) with sequential radiation therapy (SERT). Propensity score matching (PSM) was employed to ensure balanced comparison between these regimens.

**Methods:**

We assessed the efficacy of P+CCRT from a phase II trial and P-GEMOX combined with SERT using real-world data. PSM was conducted at a 1:1 ratio with a caliper of 0.18 to align baseline characteristics between the treatment groups. Key outcomes analyzed included overall response rate (ORR), complete response rate (CR), progression-free survival (PFS), overall survival (OS), and AEs.

**Results:**

Following PSM, the study included 52 patients, with 26 in each treatment group. Baseline characteristics were balanced between the cohorts. The ORR for P+CCRT group was 100.0% compared to 88.5% for P-GEMOX+ SERT group, and the CR rates was 100.0% *vs*. 76.9%, respectively. The 3-year OS and PFS rates were both 92.3% for P+CCRT, while P-GEMOX showed 92.3% OS and 80.8% PFS. Adverse events, including hematological toxicity, hepatotoxicity, and coagulation dysfunction, were comparable between the two regimens.

**Conclusion:**

P+CCRT is associated with comparable clinical outcomes compared to P-GEMOX + SERT in early-stage NKTCL, with comparable adverse events. Additionally, P+CCRT offers the benefit of a more streamlined treatment regimen with a shorter cycle. Given these encouraging results, further cohort studies are needed to validate these results.

## Introduction

Natural killer/T cell lymphoma (NKTCL) arises from the mature natural killer cells and T cells. NKTCL is a pathogenic subtype of the malignant aggressive lymphoma that predominantly affects Asian and South American populations [1]. The pathophysiology of NKTCL is complex and not fully understood, though it is closely associated with Epstein-Barr virus (EBV) infection [2]. NKTCL is classified into nodal (nNKTCL) and extra-nodal (eNKTCL) forms, with eNKTCL being the more common [3].

Conventional treatments, such as anthracycline-based regimens like CHOP (cyclophosphamide, doxorubicin, vincristine, and prednisone), are often insufficient in NKTCL due to the presence of a multidrug-resistant glycoprotein that is solely produced by tumor cells [4]. Consequently, the 5-year overall survival rate of NKTCL patients is less than 50% [5]. Current treatment options for NKTCL include local radiotherapy for early-stage local lesions [5,6], as well as combinations like GELOX (gemcitabine, L-asparaginase, and oxaliplatin) [7], SMILE (dexamethasone, methotrexate, ifosfamide, L-asparaginase, and etoposide), and DDPG (pegaspargase, gemcitabine, cisplatin, and dexamethasone) [8]. These regimens may be used in conjunction with sequential or concurrent chemotherapy and radiotherapy. While these combinatorial therapies have improved patient outcomes, they often come with frequent and severe adverse effects [9,10].

Recent studies have shown that P-GEMOX followed by sequential radiation therapy is effective for early-stage NKTCL [11]. However, the high doses of chemotherapy involved can lead to significant hematological and liver function side effects [12]. Additionally, there is evidence suggesting initiating radiation therapy earlier may enhance treatment efficacy for early-stage NKTCL patients [13]. To investigate the potential benefits of earlier radiation therapy combined with chemotherapy, we conducted a phase two clinical trial assessing pegaspargase monotherapy in conjunction with concurrent radiation therapy (P+CCRT). This approach aimed to provide a simpler and more effective treatment option for patients with early-stage NKTCL. This trial demonstrated that the P+CCRT regimen was highly effective, with all patients achieving complete remission upon completion of the therapy and experiencing relatively few treatment-related side effects [14].

In our study, combined with SERT served as the control group, while P+CCRT was the experimental group. We employed a 1:1 propensity score matching method, creating 26 matched pairs for a total of 52 patients. The primary objective was to compare the response rates and adverse events of these two regimens, aiming to provide insights for optimizing treatment strategies for patients with NKTCL.

## Method and Materials

### Subjects and interventions

The study is a retrospective analysis based on a previously conducted phase two clinical trial, which included 30 untreated patients with early stage NKTCL between January 2016 and August 2019 [14]. For this analysis, we matched these patients 1:1 with individuals who received the P-GEMOX regimen followed by sequential radiotherapy at our facility, with diagnoses ranging from June 2007 to October 2020. To ensure comparability between the two groups, we applied identical inclusion and exclusion criteria. Inclusion criteria were as follows: biopsy-confirmed early-stage NKTCL (Ann Arbor stage IE or IIE) ENKTL, untreated, age 18 years or older, with an expected survival time >3 months, Eastern Cooperative Oncology Group (ECOG) performance status score of 0 to 2, and adequate hematological (absolute neutrophil count >1.5 × 10^9^/L, platelet count > 80 × 10^9^/L), renal (serum creatinine ≤1.5 mg/dL, creatinine clearance ≥50 mL/min), and liver function (aminotransferase ≤2.5 × upper limit of normal [ULN], total bilirubin ≤1.5 × ULN). Exclusion criteria included any history or concurrent malignancies and medical conditions that could affect compliance with the study protocol. The research received approval from the Ethics Committee of Sun Yat-sen University Cancer Center (Approval number: B2018-053-01), and all participants provided written informed consent in accordance with the principles of the Declaration of Helsinki.

For the P-GEMOX +SERT treatment, pegaspargase 2000 IU/m^2^ was given intramuscularly on day 1; gemcitabine 1000 mg/m^2^ was administered intravenously on days 1 and 8; and oxaliplatin 130 mg/m^2^ was infused on day 1, with cycles lasting 21 days. The number of cycles was determined by the physician based on patient characteristics and response. Radiation therapy followed chemotherapy, with a total dose of 50.0 Gy. For the P+CCRT treatment, patients received radiation concurrently with two cycles of pegaspargase (2500 units/m^2^ every three weeks), followed by four additional cycles, totaling six cycles, with the radiation dose also at 50 Gy. Details of the comparison of the two regimens are presented in Table A1. Adverse events were graded using the National Cancer Institute Common Terminology Criteria for Adverse Events version 5.0.

### Propensity score matching and Endpoints

Due to the limitations of randomizing participants in this retrospective observational study, we employed propensity score matching (PSM) to create 1:1 pairs of patients, aiming to eliminate statistical differences between the two treatment groups. A caliper of 0.18 was applied for this matching process [15,16]. To enhance comparability, we only included 28 patients from our center and excluded 2 patients from other centers. The baseline characteristics used for matching include age, sex, B symptoms, ECOG performance status, Ann Arbor stage, primary site, local infiltration, lymph node involvement, extranodal involvement, bone marrow involvement, serum lactate dehydrogenase, serum albumin concentration, lymphocyte count, hemoglobin concentration, and platelet count. The primary endpoints include ORR, CRR, and secondary endpoints include OS, PFS, the assessment of AEs associated with each treatment regimen. The Lugano criteria from 2014 were used to evaluate the response rate [17].

### Statistical analysis

We utilized the chi-square test to examine differences in dichotomous variables between the groups. PFS was calculated from the date of diagnosis to the date of disease progression, relapse, death, or last follow-up, whichever occurred first. OS was measured from the date of diagnosis to the date of death due to any causes. PFS and OS were analyzed by using the Kaplan-Meier method to plot survival curves and employed the log-rank test to compare survival differences between different groups. We used SPSS software version 25.0 (IBM Corp., Armonk, NY, USA) for statistical analysis and R version 4.1.0 (R Foundation for Statistical Computing, Vienna, Austria) for propensity score matching and data visualization. A *p*-value of less than 0.05 was considered statistically significant.

## Results

### Basic clinical characteristics of the patients

**Figure 1:**
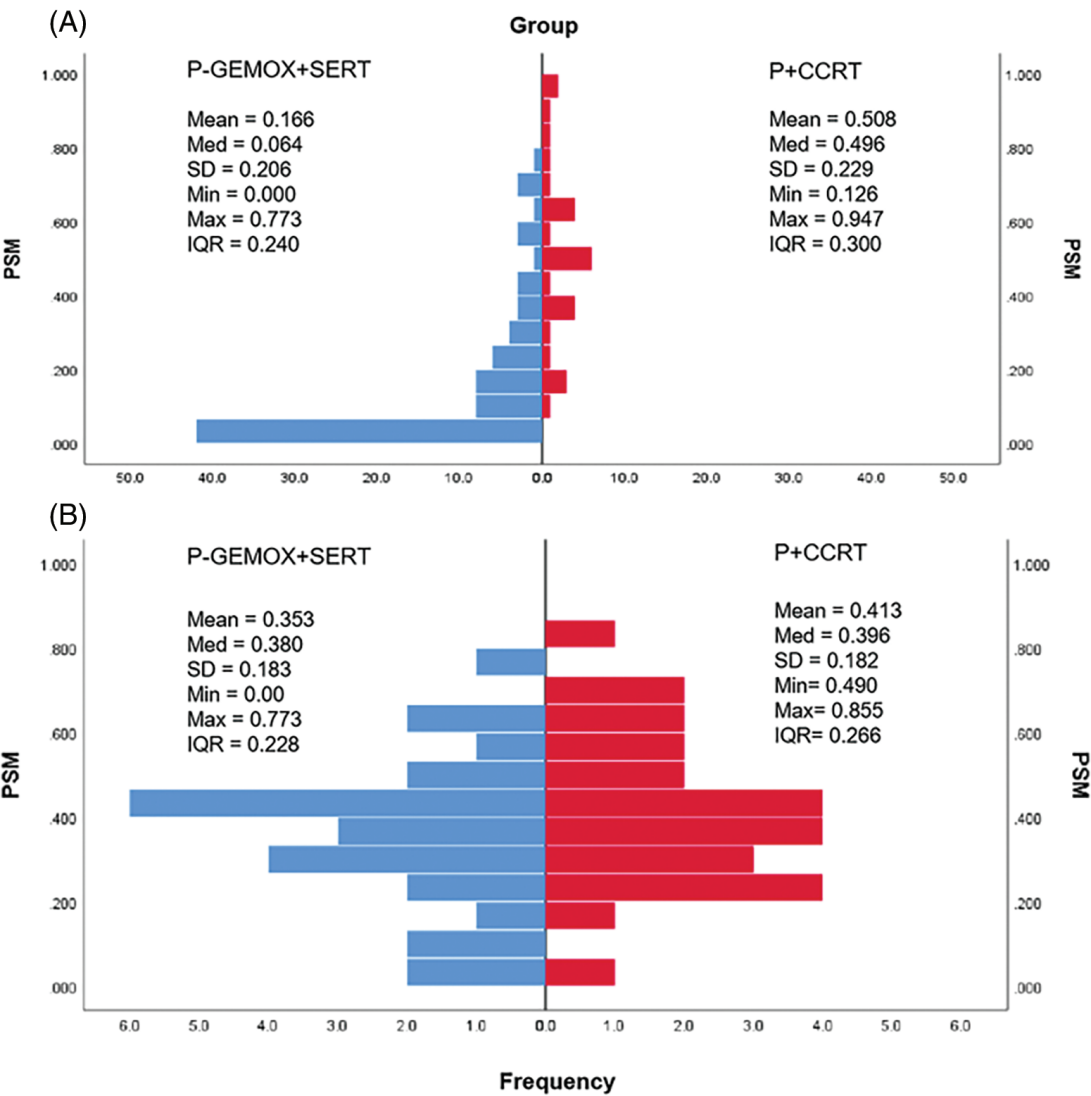
Histograms of the propensity score distribution in two groups. (A) the distribution before matching; (B) the distribution after matching. P-GEMOX, pegaspargase, gemcitabine, oxaliplatin; SERT, sequential radiation therapy; P+CCRT, pegaspargase with concurrent radiation therapy; PSM, propensity score matching; Med, median; SD, standard deviation; Min, minimum; Max, maximum; IQR, interquartile range.

Fig. 1 illustrates the distribution of propensity score for both the P-GEMOX and P+CCRT groups before and after matching. Tables 1–2 present the baseline clinical features of the patients before and after PSM. After applying PSM, 52 patients were included, with 26 in each group. In the P-GEMOX+SERT and P+CCRT groups, there were 2 and 3 patients over 60 years old, 12 and 18 with B symptoms, 5 and 10 at Ann Arbor stage II, and 21 and 16 at Stage I, respectively. Additionally, in the P-GEMOX+SERT group, 1 patient had a primary site was outside the nasal cavity, while all patients in the P+CCRT group had their primary site in the nasal cavity. Post-matching analysis using the chi-square test showed no statistically significant differences in the initial clinical features between the two groups. The characteristics of 83 patients from the real-world are presented in Table A2.

**Table 1:** Baseline clinical characteristics of patients before propensity score matching

Characteristics	P-GEMOX+SERT (n = 83, %)	P+CCRT (n = 28, %)	*p*
Age			1.000
≤60	71 (85.5%)	24 (85.7%)	
>60	12 (14.5%)	4 (14.3%)	
Sex			0.500
Male	53 (63.9%)	20 (71.4%)	
Female	30 (36.1%)	8 (28.6%)	
B symptoms			0.375
Absence	47 (56.6%)	19 (67.9%)	
Presence	36 (43.4%)	9 (32.1%)	
ECOG performance status			0.404
0/1	69 (83.1%)	21 (75.0%)	
≥2	14 (16.9%)	7 (25.0%)	
Ann arbor stage			0.062
I/II	72 (86.7%)	28 (100%)	
III/IV	11 (13.3%)	0 (0%)	
Primary site			0.006
Nasal	65 (78.3%)	28 (100%)	
Non-nasal	18 (21.7%)	0 (0%)	
Local invasion			0.650
No	28 (33.7%)	11 (39.3%)	
Yes	55 (66.3%)	17 (60.7%)	
Lymph-node involvement			0.144
None	38 (45.8%)	16 (57.1%)	
Regional	29 (34.9%)	11 (39.3%)	
Distant	16 (19.3%)	1 (3.6%)	
Extranodal involvement			0.795
0 or 1	65 (78.3%)	21 (75.0%)	
≥2	18 (21.7%)	7 (25.0%)	
Bone-marrow involvement			0.570
No	80 (96.4%)	28 (100%)	
Yes	3 (3.6%)	0 (0%)	
Initial SUVmax			0.346
≤12.1	25 (30.1%)	6 (21.4%)	
>12.1	30 (36.1%)	8 (28.6%)	
Unknown	28 (33.7%)	14 (50.0%)	
Epstein-Barr virus DNA in blood			0.553
Detectable	49 (59.0%)	13 (46.4%)	
Non-detectable	20 (24.1%)	9 (32.1%)	
Unknown	14 (16.9%)	6 (21.4%)	
Serum lactate dehydrogenase			0.795
Normal	65 (78.3%)	21 (75.0%)	
Increased	18 (21.7%)	7 (25.0%)	
Serum albumin concentration			0.457
≤35 g/L	21 (25.3%)	5 (17.9%)	
>35 g/L	62 (74.7%)	23 (82.1%)	
Lymphocyte count			0.426
≤1000 per mm^3^	16 (19.3%)	8 (28.6%)	
>1000 per mm^3^	67 (80.7%)	20 (71.4%)	
Haemoglobin concentration			0.297
≤100 g/L	9 (10.8%)	1 (3.6%)	
>100 g/L	74 (89.2%)	27 (96.4%)	
Platelet count			0.328
≤75,000 per mm^3^	5 (6.0%)	0 (0%)	
>75,000 per mm^3^	78 (94.0%)	28 (100%)	

Note: The *p*-value comparing P-GEMOX+SERT group and P+CCRT group was calculated via the chi-square test.

**Table 2:** Baseline clinical characteristics of patients after propensity score matching

Characteristics	P-GEMOX+SERT (n = 26, %)	P+CCRT (n = 26, %)	*p*
Age			0.668
≤60	24 (92.3%)	23 (88.5%)	
>60	2 (7.7%)	3 (11.5%)	
Sex			0.548
Male	17 (65.4%)	19 (73.1%)	
Female	9 (34.6%)	7 (26.9%)	
B symptoms			0.095
Absence	12 (46.2%)	18 (69.2%)	
Presence	14 (53.8%)	8 (30.8%)	
ECOG performance status			0.726
0/1	22 (84.6%)	20 (76.9%)	
≥2	4 (15.4%)	6 (23.1%)	
Ann arbor stage			
I	21 (80.8%)	16 (61.5%)	0.126
II	5 (19.2%)	10 (38.5%)	
Primary site			1.000
Nasal	25 (96.2%)	26 (100%)	
Non-nasal	1 (3.8%)	0 (0%)	
Local invasion			0.404
No	14 (53.8%)	11 (42.3%)	
Yes	12 (46.2%)	15 (57.7%)	
Lymph-node involvement			0.664
None	16 (61.5%)	15 (57.7%)	
Regional	8 (30.8%)	10 (38.5%)	
Distant	2 (7.7%)	1 (3.8%)	
Extranodal involvement (n)			1.000
0 or 1	20 (76.9%)	20 (76.9%)	
≥2	6 (23.1%)	6 (23.1%)	
Bone-marrow involvement			
No	26 (100%)	26 (100%)	
Yes	0 (0%)	0 (0%)	
Serum lactate dehydrogenase			1.000
Normal	20 (76.9%)	20 (76.9%)	
Increased	6 (23.1%)	6 (23.1%)	
Serum albumin concentration			0.726
≤35 g/L	5 (19.2%)	4 (15.4%)	
>35 g/L	21 (80.8%)	22 (84.6%)	
Lymphocyte count			0.743
≤1000 per mm^3^	5 (19.2%)	7 (26.9%)	
>1000 per mm^3^	21 (80.8%)	19 (73.1%)	
Haemoglobin concentration			1.000
≤100 g/L	0 (0%)	0 (0%)	
>100 g/L	26 (100%)	26 (100%)	
Platelet count			
≤75,000 per mm^3^	0 (0%)	0 (0%)	
>75,000 per mm^3^	26 (100%)	26 (100%)	

Note: The *p*-value comparing P-GEMOX group and P+CCRT group was calculated via the chi-square test.

### Treatment outcomes and prognostic factors

Table 3 details the treatment outcomes for both groups. In the P-GEMOX+SERT group, 20 patients (76.9%) achieved CR and 3 patients (11.5%) had a partial response (PR), resulting in an ORR of 88.5%. In contrast, all 26 patients in the P+CCRT group achieved complete response (CR) by the end of treatment. The median follow-up time was 126 months (95% CI: 99–152 months) for the P-GEMOX+SERT group and 54 months (95% CI: 46–62 months) for the P+CCRT group. To account for the differences in follow-up duration, Kaplan-Meier (K–M) curves were standardized to 60 months. The 3-year OS and PFS rates in the P+CCRT group were both 92.3% (95% CI: 82.6%–100%), compared to 92.3% (95%CI: 82.6%–100%) and 80.8% (95% CI: 67.0%–97.4%) in the P-GEMOX+SERT group, the *p-*values for OS and PFS comparisons were 0.893 and 0.473, respectively (Table 4).

**Table 3:** Comparison of the therapeutic effects of the two treatment regimens

Response	Number of patients (%)		
	P-GEMOX+SERT	P+CCRT	*p*
	*N* = 26	*N* = 26	
CR	20 (76.9)	26 (100)	0.648
PR	3 (11.5)	0 (0.0)	
SD	0 (0.0)	0 (0.0)	
PD	3 (11.5)	0 (0.0)	
ORR	23 (88.5)	26 (100)	

Note: The *p*-value comparing P-GEMOX+SERT and P+CCRT was calculated via the chi-square test.

**Table 4:** 3-years PFS and OS rate of two groups

Group	Follow-up time, Median (IQR, months)	3-y OS rate (%)	95% CI	3-y PFS rate (%)	95% CI
GEMOX+SERT (N = 26)	60 (58–60)	92.3	82.6–100	80.8	65.7–95.8
P+CCRT (N = 26)	41 (10–59)	92.3	82.6–100	92.3	82.6–100

**Figure 2:**
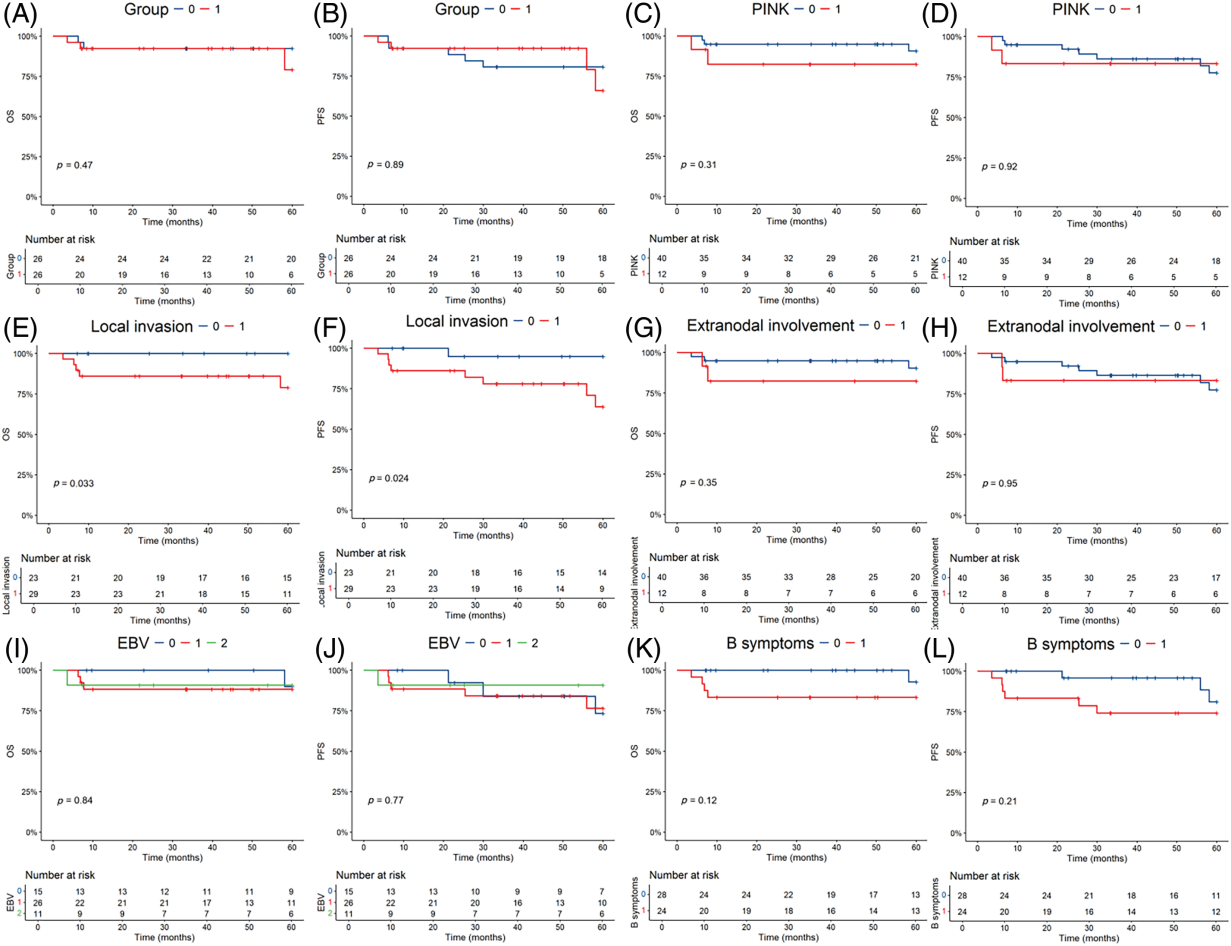
Kaplan-Meier survival analysis of the overall survival (OS) and progression-free survival (PFS) in all patients. The *p*-value reported in the figure is the result of the log-rank test. (A, B) OS and PFS stratified by the two different regimens. 0 = P-GEMOX, 1 = P+CCRT. (C, D) OS and PFS stratified by the Prognostic index for natural kill lymphoma (PINK). 0 = PINK Score is 0, 1 = PINK Score is 1. (E, F) OS and PFS stratified by the local invasion. 0 = No, 1 = Yes. (G, H) OS and PFS stratified by the extranodal involvement. 0 = No, 1 = Yes. (I, J) OS and PFS stratified by EBV DNA. 0 = Undetectable, 1 = Detectable, 2 = Unknown. (K, L) OS and PFS stratified by the B symptoms. 0 = No, 1 = Yes.

OS (Fig. 2A) and PFS (Fig. 2B) K–M survival curves revealed no statistically significant difference in the prognosis between the two groups. Among the prognostic factors, local invasion (Fig. 2E,F) was identified as a significant factor in early-stage patients. However, the prognostic index of natural killer lymphoma (PINK) score (Fig. 2C,D), extranodal involvement (Fig. 2G,H), detectable EBV DNA (Fig. 2I,J) and the presence of B-cell malignancy associated symptoms (Fig. 2K,L) did not show statistically significant prognostic value.

### Adverse reactions

Adverse reactions in both the P-GEMOX+SERT and P+CCRT groups were predominantly mild (Grades 1–2). Severe adverse reactions (Grades 3–4) were mostly related to the hematologic system, including leukopenia (15.4% *vs*. 11.5%) and neutropenia (23.1% *vs*. 23.1%), with no significant differences observed. Additionally, 11.5% of patients in the P-GEMOX+SERT group experienced Grades 3–4 anemia, whereas no such cases were reported in the P+CCRT group. Neither group experienced severe thrombocytopenia. For non-hematologic adverse reactions, most were Grades 1–2, with the P+CCRT regimen showing a higher incidence of hypoalbuminemia (*p* = 0.046). Detailed data on common chemotherapy-related toxicities for both groups are summarized in Table 5.

**Table 5:** Comparison of related adverse effects of the two treatment regimens

Toxicity	P-GEMOX+SERT (n = 26)		P+CCRT (n = 26)		*p*
	Grades 1–2	Grades 3–4	Grades 1–2	Grades 3–4	
Hematologic					
Leukopenia	13 (50.0)	4 (15.4)	16 (61.5)	3 (11.5)	0.542
Neutropenia	9 (34.6)	6 (23.1)	12 (46.2)	6 (23.1)	0.618
Anemia	10 (38.5)	3 (11.5)	9 (34.6)	0 (0.0)	0.135
Thrombocytopenia	5 (19.2)	0 (0.0)	0 (0.0)	0 (0.0)	1.000
Non-hematological adverse events					
Hypofibrinogenemia	4 (15.4)	2 (7.7)	10 (38.5)	4 (15.4)	0.204
Hypoalbuminemia	7 (26.9)	3 (11.5)	17 (65.4)	3 (11.5)	0.046
Prolonged APTT	15 (57.7)	1 (3.8)	18 (69.2)	1 (3.8)	0.685
Hyperbilirubinemia	4 (15.4)	0 (0.0)	5 (19.2)	0 (0.0)	1.000
ALT elevation	13 (50.0)	3 (11.5)	18 (69.2)	1 (3.8)	0.309
AST elevation	12 (46.2)	1 (3.8)	8 (30.8)	0 (0.0)	0.275
Nausea	15 (57.7)	1 (3.8)	17 (65.4)	0 (0.0)	0.541
Vomiting	17 (65.4)	1 (3.8)	10 (38.5)	0 (0.0)	0.077
Anorexia	13 (50.0)	1 (3.8)	14 (53.8)	0 (0.0)	0.702
Mucositis	17 (65.4)	0 (0.0)	13 (50.0)	0 (0.0)	0.278

Note: The *p*-value comparing P-GEMOX+SERT and P+CCRT was calculated via the chi-square test.

## Discussion

In this study, we have examined the potential effectiveness and adverse effects of two radiation and chemotherapy regimens in patients with early stage NKTCL. While our findings indicate that pegaspargase combined with concurrent radiation therapy may offer comparable clinical outcomes, no significant differences were observed in efficacy parameters between the two treatment groups.

Historically, basic local radiation was considered the most effective treatment for early-stage NKTCL [18]. Although some patients still experienced recurrence [19]. Similarly, anthracycline-based therapy, while commonly used, often led to relapse and low CR rates [20]. The addition of chemotherapy to radiation has markedly improved clinical outcomes for NKTCL patients [21]. For instance, earlier studies demonstrated relatively higher expression of P-glycoprotein as well as its associated mRNA in T-cell lymphoma [22]. Pegaspargase, a member of the ATP-binding cassette (ABC) transporter superfamily, has been identified as a significant factor in drug resistance for NK/T-cell lymphoma cells [23]. Its role in minimal residual lesions (MRD) also contribute to the risk of disease recurrence [24].

Pegaspargase can hydrolyze asparagine in the lymphoma cells that are unable to synthesize this essential amino acid to achieve its anti-tumor effect and was not affected by multidrug resistance of NKTCL as mentioned above, and has been reported to be effective against the NKTCL [25,26]. The polyethylene glycol covalent linkage form of pegaspargase can not only significantly prolong the half-life of the drug in the body but can also effectively reduce the chance of allergic reactions in the body [27].

We employed propensity score matching to account for baseline differences between the groups, ensuring a more accurate comparison. Our analysis demonstrated that P+CCRT achieved a significantly higher CRR of 100% compared to 76.9% in the P-GEMOX+SERT group, and ORR of 100% *vs*. 88.5%, respectively. Both regimens showed similar 3-year OS rates of 92.3%, but P+CCRT demonstrated a higher 3-year PFS rate at 92.3%, compared to 80.8% for P-GEMOX+SERT. The shorter treatment cycle and simpler administration of P+CCRT, involving intramuscular pegaspargase injections every three weeks and concurrent radiation, contributed to these improved outcomes. However, despite all patients achieving CR at the end of treatment, 1 patient (3.8%) in the P+CCRT group experienced local recurrence nine months later. This recurrence was associated with localized lesion invasion at initial diagnosis and a high Ki67 index.

In our phase II study, designed based on Simon’s optimal two-stage design, we aim to reject a 70% CR rate and support a target CR rate of 90% for early-stage ENKTL Our results surpassed this target, achieving a 100% CR rate [28]. To explore these findings, we compared the results from the phase II trial with real-world case series data.

The P+CCRT regimen, which did not involve intravenous chemotherapy, showed minimal hematology toxicity, with no grade 4 myelosuppression reported. Although pegaspargase is associated with several adverse effects such as altered coagulation function and pancreatitis [29,30]. The most severe adverse reactions in our study were grade 3 hypofibrinemia in 3 patients and grade 4 APTT prolongation in 1 patient. These were managed successfully with symptomatic treatment. No cases of pancreatitis were observed, and there was no therapy-related mortality. Both the prolonged APTT and grade four hypoalbuminemia found in the control group were not observed in the P+CCRT group.

Our study has limitations. The small sample size may affect the robustness and generalizability of our findings. Additionally, despite using propensity scores to control for observed variables, unmeasured confounding factors might still bias the results. Future research should aim to identify and mitigate the impact of these unmeasured variables to enhance the validity of the findings.

## Conclusion

In conclusion, our trial indicates that pegaspargase combined with concurrent radiation therapy may offer comparable outcomes for patients with early-stage NKTCL. While the results are encouraging, the study’s limitations highlight the need for further large-scale, multi-center studies to confirm these findings.

## Data Availability

The data used and analyzed during the current study are available from the corresponding author on reasonable request.

## Appendix A

**Table A1:** Exihibition of the two regimens

Agents	Dose	Route	Time	Cycle	Radiotherapy
P-GEMOX+SERT				- Each cycle lasts 21 days - Number of cycles determined by physician	50 Gy
Pegaspargase	2000 IU/m^2^	IM	day 1		
Gemcitabine	1000 mg/m^2^	IV	day 1 and 8		
Oxaliplatin	130 mg/m^2^	IV	day		
P+CCRT				- 2 cycles followed by 4 cycles, totaling 6 cycles	50 Gy
Pegaspargase	2500 IU/m^2^	IM	day 1		

Note: IM, Intramuscular injection; IV: Intravenous catheterization.

**Table A2:** Characteristics of real-world patients

Characteristics	Exluded (n = 57)	Included (n = 26)	*p*
Age			
≤60	47 (82.5%)	24 (92.3%)	0.397
>60	10 (17.5%)	2 (7.7%)	
Sex			
Female	18 (31.6%)	12 (46.2%)	0.225
Male	39 (68.4%)	14 (53.8%)	
B symptoms			
Absence	36 (63.2%)	11 (42.3%)	0.124
Presence	21 (36.8%)	15 (57.7%)	
ECOG			
0/1	48 (84.2%)	21 (80.8%)	0.942
≥2	9 (15.8%)	5 (19.2%)	
Ann Arbor stage			
I/II	46 (80.7%)	26 (100.0%)	0.04
III/IV	11 (19.3%)	0 (0.0%)	
Primary site			
Nasal	40 (70.2%)	25 (96.2%)	0.017
Non-nasal	17 (29.8%)	1 (3.8%)	
Local invasion			
No	15 (26.3%)	13 (50.0%)	0.062
Yes	42 (73.7%)	13 (50.0%)	
Lymph-node involvement			
None	21 (36.8%)	17 (65.4%)	0.039
Regional	22 (38.6%)	7 (26.9%)	
Distant	14 (24.6%)	2 (7.7%)	
Extranodal involvement(s)			
0 or 1	45 (78.9%)	20 (76.9%)	1
≥2	12 (21.1%)	6 (23.1%)	
Bone marrow invasion			
No	54 (94.7%)	26 (100.0%)	0.577
Yes	3 (5.3%)	0 (0.0%)	
Initial SUVmax			
≤12.1	20 (50.0%)	5 (33.3%)	0.423
>12.1	20 (50.0%)	10 (66.7%)	
Epstein-Barr virus DNA in blood			
Detectable	13 (27.1%)	7 (33.3%)	0.812
Non-detectable	35 (72.9%)	14 (66.7%)	
Serum lactate dehydrogenase			
Normal	45 (78.9%)	20 (76.9%)	1
Increased	12 (21.1%)	6 (23.1%)	
Serum albumin concentration			
≤35 g/L	42 (73.7%)	20 (76.9%)	0.966
>35 g/L	15 (26.3%)	6 (23.1%)	
Lymphocyte count			
≤1000 per mm^3^	46 (80.7%)	21 (80.8%)	1
>1000 per mm^3^	11 (19.3%)	5 (19.2%)	
Haemoglobin concentration			
≤100 g/L	49 (86.0%)	25 (96.2%)	0.315
>100 g/L	8 (14.0%)	1 (3.8%)	
Platelet count			
≤75,000 per mm^3^	52 (91.2%)	26 (100.0%)	0.289
>75,000 per mm^3^	5 (8.8%)	0 (0.0%)	
